# Heart rate monitoring to detect acute pain in non-verbal patients: a study protocol for a randomized controlled clinical trial

**DOI:** 10.1186/s12888-023-04757-1

**Published:** 2023-04-14

**Authors:** Emilie S. M. Kildal, Daniel S. Quintana, Attila Szabo, Christian Tronstad, Ole Andreassen, Terje Nærland, Bjørnar Hassel

**Affiliations:** 1 K.G. Jebsen, Centre for Neurodevelopmental Disorders, University of Oslo, Oslo, Norway; 2 Institute of Clinical Medicine, Faculty of Medicine, University of Oslo, Oslo, Norway; 3 Department of Psychiatry, Lovisenberg Diakonale Sykehus, Oslo, Norway; 4 Department of Psychology, University of Oslo, Oslo, Norway; 5 NORMENT, Division of Mental Health and Addiction, University of Oslo, Oslo, Norway; 6 NevSom, Department of Rare Disorders, Oslo University Hospital, Oslo, Norway; 7 Department of Clinical and Biomedical Engineering, Oslo University Hospital, Oslo, Norway; 8 Department of Neurohabilitation, Oslo University Hospital, Oslo, Norway

**Keywords:** Heart rate monitor, Acute pain, Non-verbal patients, Autism spectrum disorder, Health technology, Autonomic nervous system

## Abstract

**Background:**

Autism entails reduced communicative abilities. Approximately 30% of individuals with autism have intellectual disability (ID). Some people with autism and ID are virtually non-communicative and unable to notify their caregivers when they are in pain. In a pilot study, we showed that heart rate (HR) monitoring may identify painful situations in this patient group, as HR increases in acutely painful situations.

**Objectives:**

This study aims to generate knowledge to reduce the number of painful episodes in non-communicative patients’ everyday lives. We will 1) assess the effectiveness of HR as a tool for identifying potentially painful care procedures, 2) test the effect of HR-informed changes in potentially painful care procedures on biomarkers of pain, and 3) assess how six weeks of communication through HR affects the quality of communication between patient and caregiver.

**Methods:**

We will recruit 38 non-communicative patients with autism and ID residing in care homes. *Assessments*: HR is measured continuously to identify acutely painful situations. HR variability and pain-related cytokines (MCP-1, IL-1RA, IL-8, TGFβ1, and IL-17) are collected as measures of long-term pain. Caregivers will be asked to what degree they observe pain in their patients and how well they believe they understand their patient’s expressions of emotion and pain. *Pre-intervention:* HR is measured 8 h/day over 2 weeks to identify potentially painful situations across four settings: physiotherapy, cast use, lifting, and personal hygiene. *Intervention:* Changes in procedures for identified painful situations are in the form of changes in 1) physiotherapy techniques, 2) preparations for putting on casts, 3) lifting techniques or 4) personal hygiene procedures. *Design:* Nineteen patients will start intervention in week 3 while 19 patients will continue data collection for another 2 weeks before procedure changes are introduced. This is done to distinguish between specific effects of changes in procedures and non-specific effects, such as caregivers increased attention.

**Discussion:**

This study will advance the field of wearable physiological sensor use in patient care.

**Trial registration:**

Registered prospectively at ClinicalTrials.gov (NCT05738278).

## Background

Social communication deficits are among the core features of autism spectrum disorder (ASD) [1]. Symptoms and support requirements for ASD vary in form and intensity with communication deficits ranging from mild to severe [2]. Approximately 30% of people with an ASD diagnosis also have intellectual disability (ID) [3], a neurological condition that affects 1% of the population [4, 5]. For low-functioning individuals with ASD or ID, the symptoms often overlap, and clear-cut diagnosis constitute a challenge [6–8]. The diagnosis of ID involves an intelligence quotient (IQ) below 70, reduced adaptive behaviour, and occurrence of the condition before the age of 18 [1, 8]. Approximately 5% of those with ID have grade severe (IQ: 20–34) or profound (IQ < 20) and need round-the-clock supervision and help [9, 10]. Although ID can be an acquired condition, for instance due to cerebral hypoxia, ischemia or infection early in life [11–14], genetic conditions, such as a de novo mutation [15], are a common cause. Severe or profound ID often coincide with cerebral palsy that affects control of muscles involved in speech, gesticulation, and grimacing. These patients with severe ASD and ID may be unable to notify their caregivers when they are In pain [16–20] and are essentially non-communicative.

There is a need to improve health services for non-verbal patients. Despite the rights of people with disabilities being enshrined in the UN Convention from 2006 [21, 22], age of death remains lower and mortality rates higher for people with intellectual and developmental disabilities [23, 24]. Patients with comorbid ASD and ID often have complex medical needs and many cannot convey their needs to their caregivers [25]. They will typically need the equivalent of 5–10 full-time staff per year–- a major challenge both in terms of competence needs and resource use [26]. Due to the demanding and person-intensive nursing attention required, there is considerable interest in technological solutions to aid communication and participation for this vulnerable group [27, 28]. Overall, technology can improve independence, participation and quality of life among people with complex needs [29].

Sensors may be used to monitor physiological parameters and activity patterns so that individuals and their caregivers can learn about their health. As a neurally mediated phenomenon, heart rate (HR) is regarded a noninvasive window into the central nervous system [30]. Changes in HR are widely used as markers of reactivity to painful events [30–37], and clinically HR is used in neonatal pain assessment and care [31]. Pain is an inferred latent process associated with several physiological and psychological markers including changes in neural activity as measured by electroencephalogram (EEG), pupil dilation, skin conductance, HR, blood pressure, and respiration, as well as self-reported pain magnitude [38, 39]. Although pain-induced increases in blood pressure may trigger vagal baroreflexes that counteract sympathetically-mediated tachycardia; sympathetic changes are found to dominate [40], making HR a reliable non-invasive way to identify probable painful events [40–42]. Although the central nervous system, cardiovascular system, and pain are closely interrelated [43, 44], HR is not a specific indicator of pain [30]. States like depression, being tense, angry or frightened may also involve an increase in HR due to a release of stress hormones like cortisol and adrenaline. The clinical application of such reactivity is shown in studies using HR to predict acute stress in ASD [45, 46]. As a more general reactivity measure HR is promising as a tool, and when context is given, it may be useful in non-verbal patient pain-management.

We have previously shown that HR can be used as a person-specific measure of reactivity for non-verbal patients [47]. To evaluate HR as a tool to assist pain management for non-verbal patients, other well-established biomarkers of pain, such as heart rate variability (HRV) and cytokine biomarkers in blood, should be utilized. HRV is a non-invasive measure of imbalances in the autonomic nervous system with a higher variance indicating less stress [48, 49]. A growing body of psychological research has shown HRV to be a stable biomarker of prolonged pain [50], and research supports an association between HRV and emotional responses [51, 52]. Recent studies suggest that certain circulating inflammatory cytokines can be utilized as reliable biomarkers for detecting chronic pain in various diseases [53–55]. Blood biomarkers of pain include MCP-1, IL-1RA, IL-8, TGFβ1, and IL-17 [53–55]. Elevated levels of these cytokine biomarkers have been associated with experimental pain and self-rated pain, and their levels have been shown to closely follow the temporal phenomenology of pain episodes [54]. The combination of HR, HRV and cytokine biomarkers may prove a novel tool for pain assessment.

Despite the potential of this approach, the use of sensors in care for non-verbal patients is not yet widespread. Most studies on sensors for pain management and communication has been conducted among patients with an ability to communicate [48, 56–60]. Non-verbal patients are frequently left out of research studies, even though advancements in medical technology could bring significant benefits to this patient population.

The proposed study is a randomized controlled trial that seeks to reduce incidence of pain for non-verbal individuals with autism and ID through use of HR-sensors. This study aims to 1) assess the effectiveness of HR as a tool to identify potentially painful care procedures; 2) test the impact of HR-informed changes on pain biomarkers; and 3) examine the impact of six weeks of HR monitoring on the quality of communication between patients and caregivers.

## Methods

### Study design overview

**Fig. 1:**
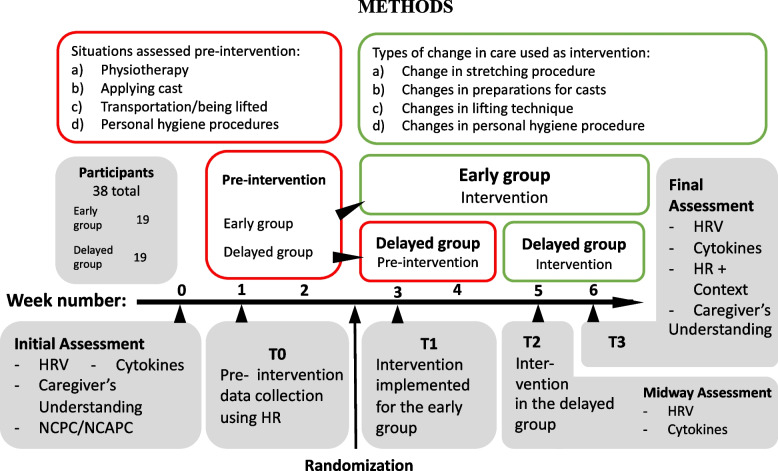
Overview of phases in a clinical study. Abbreviations: HR: heart rate, HRV: heart rate variability, NCPC: Non-communicating Children’s Pain Checklist; NCAPC: Non-Communicating Adult Pain Checklist

This is a single centre, controlled, randomized trial with 38 non-verbal participants, testing if HR-guided interventions in suspected painful settings will change the incidence of pain. The study design includes a two-week *pre-intervention*, consisting of continuous HR registering across four potentially painful settings; and an *intervention phase* consisting of formal change in procedure for the identified painful situation (Fig. 1). The interventions are in one of four forms: changes in 1) physiotherapy techniques, 2) preparations for putting on casts, 3) lifting techniques or 4) personal hygiene procedures. HRV and inflammatory cytokines (MCP-1, IL-1RA, IL-8, TGFβ1, and IL-17) are measured at the beginning, midway, and end of the study period to assess change in long-term pain. Participants will be randomly assigned to either an early intervention group or a delayed intervention group in a 1:1 ratio using a computer-generated randomization scheme. This will differentiate the effect of formal change in routine from increased attentiveness due to study participation, and instant changes made when the HR band signify obvious acute pain (such as skin pinched while putting on a cast). Overview over measures and objectives is given in Table 1.

**Table 1:** Timeline of trial illustrating the timing of different biomarkers and data collection, as well as their corresponding hypothesis

Timepoint	T0		T1		T2	
Nr. Week	1	2	3	4	5	6
Early group	Pre-intervention phase^a^		Change in care procedure			
Delayed group	Pre-intervention phase^a^				Change in care procedure	
Time of samples collected and corresponding hypothesis						
*Primary hypothesis*	HR↑ + context	HR↑ + context	HR↑ + context	HR↑ + context	HR↑ + context	HR↑ + context
*1*^*st*^* Secondary*	HR↑	HR↑	HR↑	HR↑	HR↑	HR↑
	Cytokines			Cytokines		Cytokines
	HRV			HRV		HRV
2^nd^* Secondary*	CU^b^					CU^b^

^a^Registration of HR increases in potentially painful situations in the patient’s everyday life

^b^Caregivers’ rated understanding of the patient as measured by a trial-specific survey, given in Table 4

*Abbreviations*: *TG* Treatment group, *DG* Delayed intervention group; HR↑ = Heart rate increases; Context = Contextual data; Cytokines = MCP-1, IL-1RA, IL-8, TGFβ1, and IL-17; HRV = heart rate variability; metabolites; CU = ^b^Caregiver’s rated understanding

### Objectives

The overarching aim is to generate knowledge to reduce incidence of pain in non-verbal patients’ everyday lives.

#### Primary objective

The primary objective is to evaluate how HR as measure of patient reactivity can be used to identify potentially painful care procedures that should be re-evaluated in terms of the approach taken.

Primary hypothesis:* HR increases as defined by 2 standard deviations (SD)* *increase in HR can identify potentially painful care situations in the patient’s everyday life that that should be re-evaluated (e.g., different methods for stretching of spastic limbs, different routines for transport with personal lifts).*

#### Secondary objectives

The secondary objectives are to:

1. Evaluate how HR-informed changes in potentially painful procedures affect pain biomarkers.

*Secondary hypothesis 1: HR-informed changes in patient-specific care procedures (a. physiotherapy, b. applying cast for spastic limbs, c. transportation/being lifted, or d. routines for personal hygiene) will increase HRV and reduce pain-related cytokines (MCP-1, IL-1RA, IL-8, TGFβ1, and IL-17) as evaluated at the beginning, mid-way and at the end of the study period.*

2) Assess how six weeks of communication through HR affects the quality of communication between patient and caregiver.

*Secondary hypothesis 2: HR as an aid in communication will increase caregivers’ understanding of the patient as measured by changes in perceived understanding of the patient from the beginning to the end of the study period.*

### Study setting

The study is conducted across several sites in Oslo and the surrounding area (Table 2), with interventions administered at care homes with round-the-clock staff or one-to-one staffed school/daycare. The project management is set in Oslo. We are creating our unique panel of patient representatives, including representatives from LUPE (national user organization for persons with ID, https://www.lupeorg.no/) and the Norwegian Cerebral Palsy Association. The patient representative group will be a national collaboration. The scientific advisory board consist of international researchers working in the field of developmental disability.

**Table 2:** Overview research group organization

Recruitment sites ^a^	Neurohabilitation, Oslo University Hospital Neurohabilitation, Ahus Hospital Nic Waals Institute, Lovisenberg Hospital District Nordstrand, Oslo Municipality District Vestre Aker, Oslo Municipality Emma Hjort, Bærum Municipality Frambu, Competence Centre for Rare Diagnoses
Project management group	University of Oslo: -Principal Investigator B. Hassel (prof.); Oslo University Hospital, Oslo, Norway; -C. Tronstad (Dr. Philos) KG. Jebsen Centre for Neurodevelopmental Disorders: -T. Nærland (Ph.D.); -E. Kildal (PhD-cand) -O. Andreassen (prof.); -D. Quintana (PhD); -A. Szabo (PhD)
Scientific advisory board^b^	Oslo Metropolitan University: C. Morland (PhD) Oslo University Hospital: S. Hope (PhD) Rigshospitalet Copenhagen: A. Sabers
Patient representatives^c^	Bærum Municipality: Chief of care, H. Hesselberg LUPE (national user organization for persons with ID): H. Kvame Cerebral Palsy organization: E. Buschmann

^a^Sites that have shown interest at start of recruitment per October 2022. ^b^Collaborators within the field, contributing to the quality of the trial and analysis of data. ^c^ Representatives, contributing with patient perspective on trial execution and with valuable insights on interpretations of findings

### Study design

#### Intervention

The intervention comprises individualized routines to reduce pain in potential pain-inducing settings.

In the pre-intervention phase, HR is continuously collected for 8 h/day across potentially painful care situations. The primary caregiver will report on situations with an HR increase (Figs. 4 and 5).

As the caregiver is not blinded to the HR increases during the registration phase, we expect instantaneous changes made when the HR band communicated obvious acute pain (e.g. skin pinched while putting on cast). These instantaneous, non-formalized changes made by each caregiver is not regarded as “change in routine” but are registered as HR-informed adaptations. The intervention consists of a formal change in routine, and involved changes is written routine and training of staff for the chosen situation. Allocation into an early group and a delayed group helps distinguish the non-formal adaptations based on HR from the effect of formal changes in procedures of care.

Based on the two-week pre-intervention phase, one of four types of care procedures are chosen as the intervention target: 1) physiotherapy, 2) applying cast on spastic limb, 3) transportation/being lifted and 4) personal hygiene procedures. If there are several situations identified as candidates for intervention, selection will be based on a) level of reproducibility and b) magnitude of HR increase. The procedure must be easily recognizable. The situation should at have an HR-spike occurrence for at least 10 episodes during the recording period (i.e. on average 1 occurrence/day), with at least 80% of the situation resulting in HR-increase (reproducibility).

The intervention will be in one of four forms:

1. changes in physiotherapy, e.g., less rigorous movement in the identified painful stretch,
2. preparations for putting on corrective cast to stabilize joint and/or stretch spastic muscles,
3. change in procedures for transportation/lifting, e.g., new technique or adjustments made to equipment, or
4. revised personal hygiene procedure.

If none of the predefined interventions fit the chosen stressor, a fitting intervention is constructed and will be described separately and in detail when the results of this study are published.

### Participants

#### Study Population

Patients with communication difficulties, here defined as an impairment in the ability to convey concepts of both verbal and nonverbal form and that the impairment is to such an extent that it causes caregivers to worry that the patient may experience pain and distress without being able to notify them.

#### Inclusion criteria

**Fig. 2:**
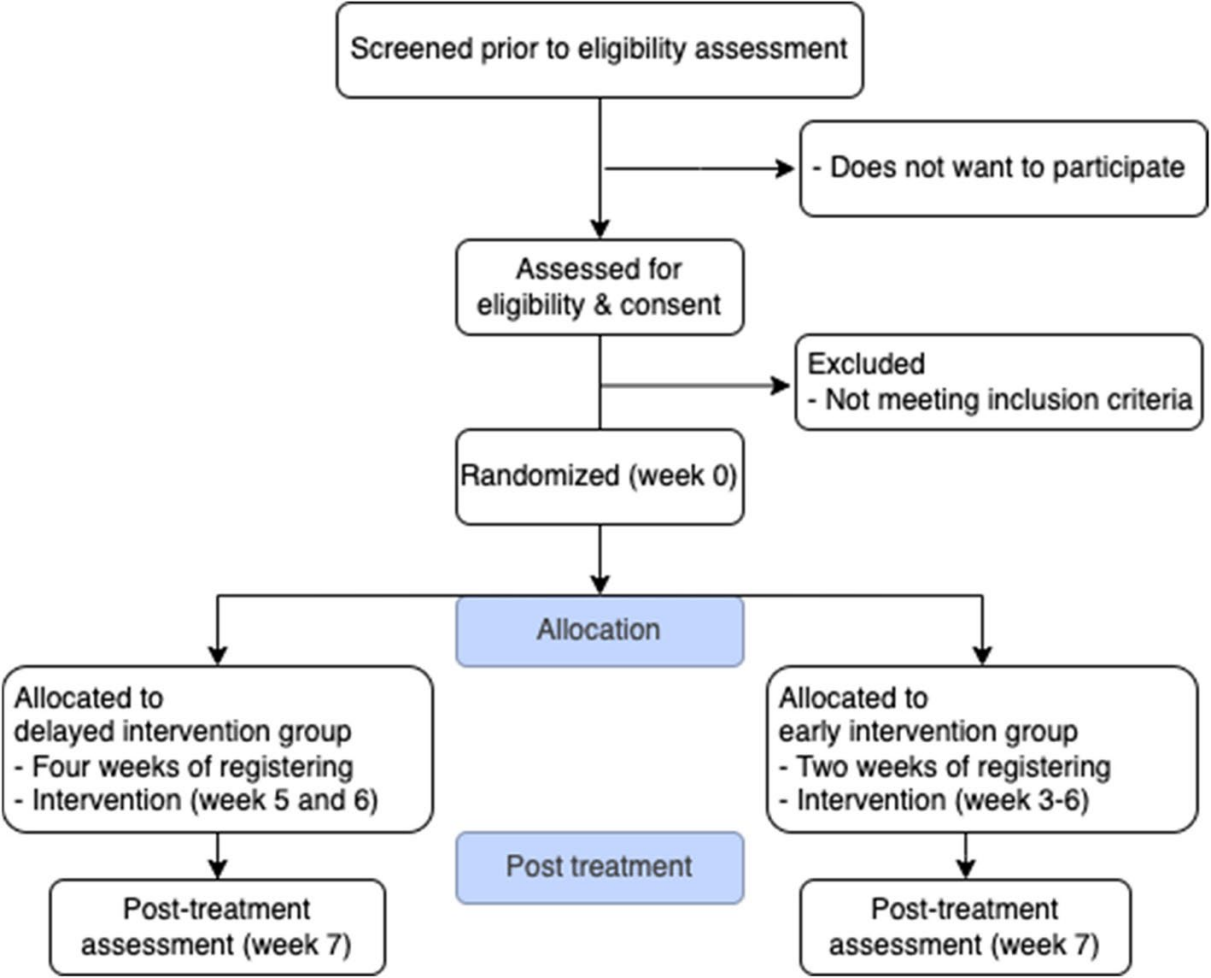
Trial flow diagram.As the participant cannot give informed consent, it is the legal representative or parent who decides on participation or to withdraw. Screening includes diagnosis of severe or profound ID and fulfilling ASD diagnostic criteria. Assessments will take place at baseline (week 1), end of week 3 and post-treatment

To be included in the study participants must meet the following criteria: Male or female between 5 and 70 years of age at the time of study inclusion. All participants will be evaluated for ASD diagnosis in accordance with ICD-10 by a clinical psychologist taking into consideration that symptoms of ASD and ID often overlap in low-functioning individuals with, making clear-cut diagnosis difficult [6–8] (Fig. 2). Social Communication Questionnaire [61–63] will be used as part of the diagnostic evaluation. Participants may have cerebral palsy to a degree that compounds the patient’s communication problems. Written informed consent must also be obtained from the participant's legal representative.

#### Exclusion criteria

Not living in a care home with round-the-clock staff for at least five days a week; or not attending one-to-one staffed school/day-care at least five days a week. Having any ongoing infection with a C-reactive protein (CRP) > 20 or any type of cancer with ongoing chemotherapy.

#### Sample size

**Fig. 3:**
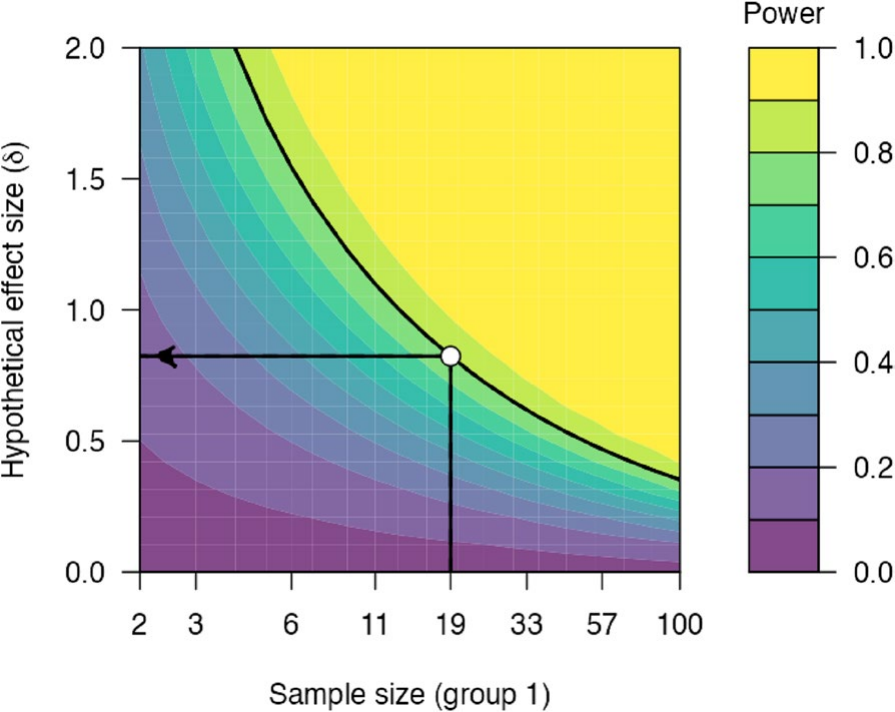
A power contour plot. Power analysis suggests that a paired samples t-test including 38 participants will reliably detect (power = 80%) an effect size of 0.82, or higher. Statistical power is also shown by assuming smaller or larger hypothetical effect sizes. Analysis and visualisation were performed using the ‘jpower’ JAMOVI module: https://github.com/richarddmorey/jpower

There are various approaches for calculating sample size [64]. Here we have decided to design the study to reliably detect our effect size of interest. We will recruit 38 patients, allowing for a dropout of 15%. With 38 patients, a one-sided paired samples t-test can reliably detect (80% power) an effect size of *d* = 0.84, alpha = 0.05 (Fig. 3). We have chosen a one-sided test as we have a directional hypothesis. This is a realistic effect size of interest given that heart rate increases in response to stressors are associated with effect sizes larger than *d* = 1 [65].

#### Recruitment

Participants are recruited via Oslo University Hospital, Ahus Hospital, Oslo-, Bærum- and Drammen municipalities, which are located in south-eastern Norway (Table 2). We will invite back people with communication difficulties who have already participated in previous phases of the project two years ago [47], as well as new participants. The investigators will present the study to the local municipalities’ administrators, who will be encouraged to contact the leaders of the districts’ communal residences for persons with ID. Professional caregivers at the care homes will approach the parents or legal representatives of potential participants with written materials describing the study. All recruitment channels will direct the interested individuals to the investigators, and written consent will be obtained from the parents or legally authorized representatives of the participants.

#### Method of assigning participants to treatment groups

The study design employs an early and a delayed intervention group to distinguish the effects of formal routine changes from increased attentiveness due to study participation and effects of instantaneous changes in care. The early intervention group will start intervention in week three while the delayed group will continue data collection for another two weeks before introducing procedure changes. Patients will be randomly assigned to one of the two groups (Fig. 1) on a 1:1 ratio using a computer-generated randomization scheme.

#### Blinding

The identity of test and delayed intervention treatments will not be known when analysing data on the secondary outcome measure. Access to the randomization code will be strictly controlled.

### Data collection

#### Clinical assessments

Demographic information (date of birth, gender) will be recorded at inclusion. Relevant medical history, including history of current disease, and information regarding comorbid diagnosis and known aetiology will be recorded at inclusion through conversation with legal representative and primary caregiver, as well as through medical records.

#### Caregivers’ reports of observed pain

Observable pain is measured using the Non-communicating Children's Pain Checklist (NCPC) for children, and Non-Communicating Adult Pain Checklist (NCAPC) for adults. NCPC is shown to be internally consistent, consistent over time, significantly related to pain intensity, sensitive to pain, and specific to pain [66]. NCAPC has shown equal reliability in assessing chronic pain in adult individuals with ID [67, 68]. Both have proved useful within pain research on participants with ID [67, 69]. Score on NCPC/NCAPC will be recorded at start of trial as this gives each participant a baseline score on how much pain is visible to caregivers prior to use of HR-monitoring.

#### HR monitoring of acute pain

**Fig. 4:**
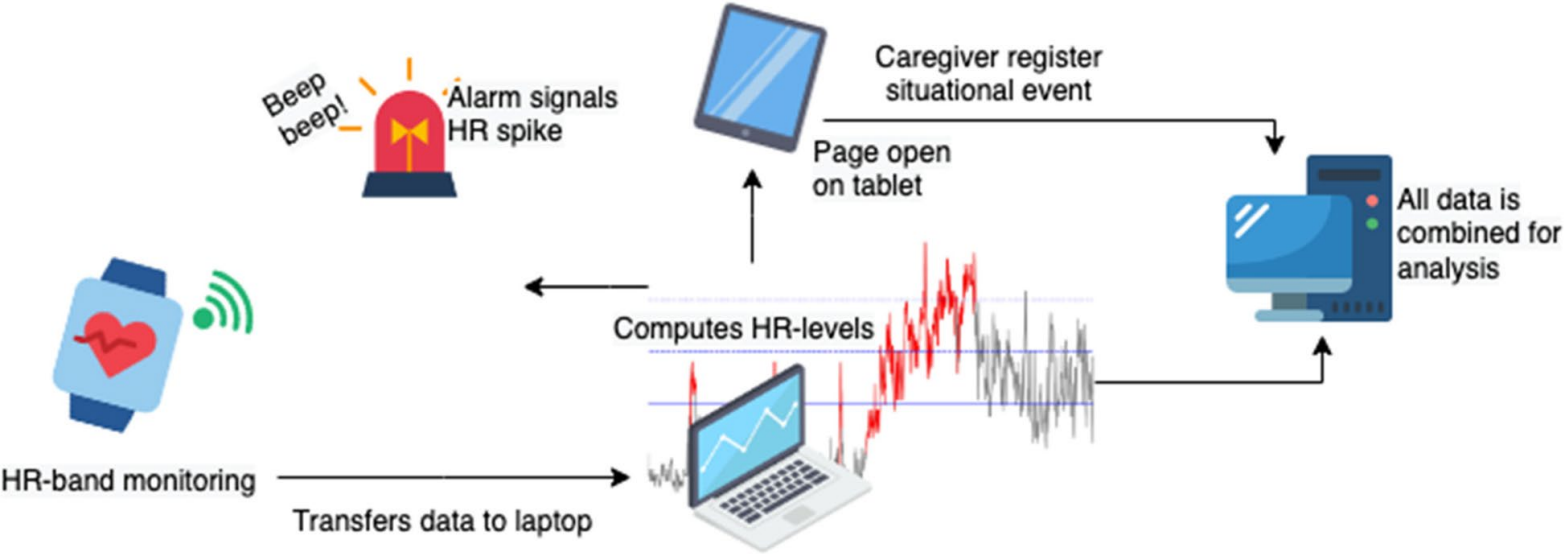
Diagram of pipeline. The workflow starts with “HR-band monitoring” registering data, then processing, alerting caregivers, and gathering sensor and situational data for analysis. The final step is the interpretation and eventual publication of results. Abbreviations, HR: heart rate. Icons from flaticon.com

Polar OH1 or Verity Sense armbands are used to calculate measures of HR [70–73]. The armband detects pulse at the upper arm (brachial artery), which is transmitted to a Microsoft Surface Pro research laptop (Fig. 4).

The laptop has a program that calculates whether the HR has risen substantially. This is done by calculating the average HR for the last 20 min. The program then calculates a measure for the variation of the HR within 20 min. If the HR increases by more than 2 standard deviations above the average HR, it sends a signal to alert the caregiver.

#### Contextual data collection

The research laptop has a registration page for providing contextual data. When an HR increase is detected, the page will automatically appear and ask for information to be provided.

**Fig. 5:**
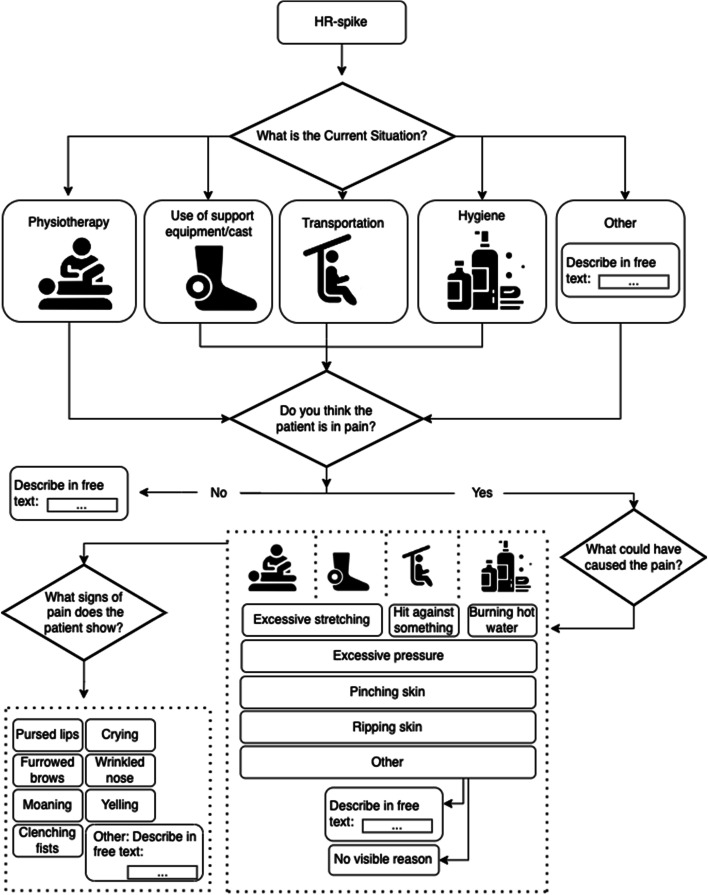
Context data collection. Presumed triggers for the increased HR is categorized and reported in the computer program during the pre-intervention phase. The caregivers will always have the possibility of reporting freely under “other”. Icons from flaticons.com

The caregiver will be given four main categories: physiotherapy/stretching, putting on/taking off cast, personal lift use, and hygiene; in addition to “other” with a free text description (Fig. 5). The caregiver checks off stressors according to a pre-made selection of psychological triggers (e.g., excessive stretching, pinched skin, and hot water). Finally, the caregivers check off what signs of pain the patient did show. On all levels there is the possibility of selecting “other” to write in free text.

#### HRV monitoring of prolonged pain

HRV is a non-invasive measure of parasympathetic nervous system activity [74, 75]. Lower levels of HRV (i.e., reduced parasympathetic nervous system activity) have been associated with both poor somatic and psychiatric health [76, 77]. Like the HR, HRV is calculated continuously through the Polar OH1 armband. Additionally, HRV will be assessed at rest with a portable electrocardiogram (ECG). The ECG will be collected at baseline, after two weeks of intervention in the early group, and at the end of the study period.

#### Biochemical measurements

We will collect blood and saliva samples to measure biomarkers for long-term pain and stress at baseline, after two weeks of intervention in the early group, and at the end of the study period.

Determinations for assessment of systemic evidence of inflammation include CRP and ferritin [78], as well as Nam [79, 80]. CRP and Nam will be analysed using marker-specific salivary kits. Blood samples will be analysed at Oslo University Hospital for haemoglobin, haematocrit, red blood cell count, white blood cell count, white blood cell differential, platelet count, erythrocyte sedimentation rate, CRP and ferritin.

Pain-related inflammatory biomarkers include MCP-1, IL-1RA, IL-8, TGFβ1, and IL-17 [81–88]. Analyses will be performed at the Department of Clinical Chemistry, Oslo University Hospital, Oslo, Norway, on an Integra 800 instrument following standard protocol Roche Diagnostics, IN, USA; for details of the diagnostics pipeline, please see Szabo et al., 2022 [89].

PhD A. Szabo will perform all analysis of inflammatory markers at Oslo University Hospital.

#### Caregivers’ reports of understanding

The participating caregivers will answer an electronic questionnaire on their understanding of the patient and how the caregivers’ level of uncertainty (Table 3). After the study, the caregivers will answer how HR monitoring has affected their understanding of the patient.

**Table 3:** Perceived understanding of the patient, questionnaire^a^

**Questions given at beginning and end of study period**	Predicted change at end of study period
Factor 1: The caregiver’s understanding of the patient	
When the patient is in pain or feels discomfort, I notice it	**↑**
I notice when the patient is happy	**↑**
When the patient is angry or irritated, I notice it	**↑**
I notice it when the patient is afraid	**↑**
I notice when the patient is sad	**↑**
Factor 2: The patient's ability to express	
The patient can tell me that he/she feels pain or discomfort	**↑**
The patient can tell me that he/she is happy	**↑**
The patient can tell me that he/she is angry	**↑**
The patient can tell me that he/she is afraid	**↑**
The patient can tell me that he/she is sad	**-**
Factor 3: The caregiver's emotional experiences	
I feel unsure whether I have understood the user	**↓**
I feel a sense of mastery because I understand the user	**↑**
I feel stressed because I don't understand the user	**↓**
I feel fear because I don't understand the user	**↓**
**Questions only included in survey given at the end of the study**	
Relative to before this study, how has your communication with the patient changed? Relative to before this study, how has your way of working with the patient changed? How satisfied are you with using HR as a means of communication? How satisfied are you with using the program for situational analysis? In general, how satisfied are you overall with system used in the trial? How often did you experience the “beep” of the HR increase to be useful, and how often not to be? What is the biggest roadblock for continuing use of the system in this study?	

^a^The questionnaire was developed in the autumn of 2020, in collaboration with B. Hassel, J. & J. Frantsen. The questionnaire was based on a review of relevant literature, our own experience, and through discussion within the research group. The questionnaire was tested in a pilot study with 17 participants to perform an exploratory factor analysis. The modified questionnaire was distributed to 135 professional caregivers in 2021, and formed the basis of Kildal et al., 2023 (in preparation). Each item in factor 1, 2 and 3 is answered on a Likert scale ranging from “never” to “always”. The hypothesis is that the sum score for questions on communication will increase after HR use

Questions only included in survey given at the end of the study are based on surveys for health technology satisfaction [91]

The questionnaire is trial specific and based on a review of relevant literature, experience working with the ID population, and discussion within the research group. The questionnaire was tested in a pilot study with 17 participants to perform an exploratory factor analysis for improved psychometric properties. The then-modified questionnaire was distributed to 135 professional caregivers in 2021 [90]. Confirmatory factor analysis indicates good model fit [90].

### Strategies to improve adherence to interventions

#### Measures of treatment compliance

Professional caregivers will be asked to what extent they, due to unplanned events, ended up missing registrations or deviating from the intervention protocol. Before every shift, the caregiver will have to sign a compliance to the intervention protocol, which also operates as a reminder for registrations. The leading caregiver will remind staff of the protocol at every shift meeting. Furthermore, all HR-increases are recorded automatically, and in addition the caregiver will be probed for contextual data for each HR-increase. The discrepancy between number of HR increases and number of contexts recorded gives us a measure of caregiver responsiveness.

### Data collection and management

#### Data management procedures

Participants will not be identified by name in the study database or on any study documents to be collected but will be identified by a site number, participant number, and initials. The principal investigator (Table 2) is responsible for all information collected on participants enrolled in this study. All data collected during this study must be reviewed and verified for completeness and accuracy by the principal investigator.

The data will be entered into Services for sensitive data (TSD): https://www.uio.no/english/services/it/research/sensitive-data/, an enclosed electronic storage system at the University of Oslo. All access to this database are strictly regulated. Only de-identified data will be shared with project team.

All procedures for the handling and analysis of data will be conducted using good computing practices meeting guidelines for the handling and analysis of data for clinical trials.

#### Data quality control and reporting

All changes to the study database will be documented.

#### Archival of data

The database is safeguarded against unauthorized access by established security procedures; appropriate backup copies of the database and related software files will be maintained. Databases are backed up by the database administrator in conjunction with any updates or changes to the database.

#### Statistical methods and considerations

Statistical analyses are performed using SPSS version 25 [92] and R Studio [93].

#### Demographic and baseline characteristics

The following demographic variables at screening will be summarized: gender and age, height, and weight (for the calculation of body mass index). Descriptive analyses will consist of means and SD for continuous variables and percentages for categorical variables.

#### Analysis of primary objectives

We hypothesize that a 2 SD increase in HR can identify potentially painful care situations that require re-evaluation. To determine whether HR monitoring is effective in identifying situations that require changes in care, we will compare the mean HR spike counts between situations that are suitable for adjustments in care versus situations that are not. If Shapiro–Wilk test show normality of data distribution, we will use a one-tailed paired sampled t-tests; if non-normality is indicated we will use the Wilcoxon signed-rank test.

The potential for HR to identify potentially painful care procedures will also be shown as descriptions of identified situations and shown as a number count (primary hypothesis). All situations identified by 2 SD increase in HR will be described and reported as count, including an overview of number of times each of the different situations were accompanied by increases in HR (reproducibility); number of HR increases with no registration (responsiveness of caregiver); number of HR increases recorded in situations not previously thought to entail pain or distress (novel discoveries); number of situations discovered through HR-use during the registration phase where instantaneous changes were made due to obvious acute pain (e.g. skin pinched while putting on cast; instantaneous clinical effect of HR); and number of situations identified that were available for adjustments in care and systematic evaluation (formal clinical applicability of HR).

#### Analysis of secondary objectives

To test evaluate the effect of using HR in improving care procedures and reducing pain, one-tailed paired samples t-tests are used to test for difference in HRV and cytokine biomarkers (MCP-1, IL-1RA, IL-8, TGFβ1, and IL-17) before and after intervention (secondary hypothesis 1). Cohen’s *d* values [94] will also be calculated to determine the size of the effect. To evaluate the effect of HR-informed changes in procedures, differences in biomarker scores across the early and delayed groups will be analysed using Wilcoxon Signed-rank test, which can evaluate whether the difference scores from each group are not equal (i.e., that they are significantly different). Critical P-values for the five cytokine measurers will be corrected for multiple comparisons using a 5% false discovery rate [95].

To test for significant change in rates of understanding the patient throughout study period (secondary hypothesis 2), we will perform one-tailed paired sampled t-tests. Additionally, Spearman’s rank-order correlation (*r*_s_) is used to investigate relationships between HR measures and caregivers report on NCPC and caregivers report on perceived understanding of the patient.

#### Exploratory post-hoc analysis

With the goal of further developing the technology of the HR-alarm and software, we will do exploratory analysis on how settings of alarm thresholds in the post-processing scripts compare with the event logs.

#### Administrative, ethical, regulatory considerations

The study will be conducted according to the Declaration of Helsinki.

Minor pain might occur as part of the project; this includes from possible skin reactions to the HR monitor, and pain associated with blood sample collection. For some, the HR monitor can be provocative emotionally, and some might be sensitive to new objects. Ideally, the use of the HR monitor should protect the participant from discomfort.

To maintain confidentiality, all laboratory specimens, evaluation forms, reports and other records will be identified by a coded number and initials only. All study records will be kept in a locked file cabinet and code sheets linking a patient’s name to a patient identification number will be stored separately in another locked file cabinet. Clinical information will not be released without the written permission of the participant. The investigators must also comply with all applicable privacy regulations (e.g., the Health Insurance Portability and Accountability Act of 1996, EU Data Protection Directive 95/46/EC).

#### Informed consent form

Informed consent will be obtained in accordance with the Declaration of Helsinki. A properly executed, written, informed consent will be obtained from each caregiver to enter the participant into the trial. Information should be given in both oral and written form and legal representatives must be given ample opportunity to inquire about details of the study.

#### Criteria for discontinuing or modifying allocated interventions

The participants in this study are particularly vulnerable, in that they cannot unequivocally convey how they react to a study measure, such as an HR monitor or bloodwork. It is therefore important to have a high degree of transparency. We believe that we achieve this by involving patient representatives in our project group, and all administrative levels (district director, mayor, agency manager, head of the care homes) in addition to parents, guardians, and caregivers being informed. Several of the mentioned collaborators will, to varying degrees, be involved in the study itself, and the transparency that this entail means security for the participants.

An HR monitor, whether it is attached to the wrist, ankle, or around the chest, can cause skin reactions, both allergic and mechanical. In that case, it must be removed, and an alternative location must be considered, or the use of the HR monitor must be discontinued. Some people with ID and ASD can be very sensitive to new objects. If this applies to the HR monitor, an alternative location must be considered, or the use of the heart rate monitor must be terminated.

As a suitable situation to intervene on is defined by an occurrence of at least 10 for the two-week pre-intervention period, for some participants it might be the case that no suitable situation is identified. In such case, there will be no intervention. However, the patients will be included in the final data presentation, as they shed light on in what proportion of patients HR may be useful for guiding formal change in care.

For caregivers (parents, guardians, family members, and healthcare personnel) the use of HR monitors can potentially distract from other tasks or make the time spent with the non-verbal person feel unnecessarily technical. Alternative placement or termination of the study for the patient in question must be considered against the benefits of using the HR monitor. In cases where it is doubted if caregivers due to such stress do not reliably follow the study protocol, data will be excluded.

A participant may be discontinued from study treatment at any time if the participants’ representatives, the investigators, or the caregivers feel that it is not in the participant’s best interest to continue. Parents or guardians will be able to withdraw the patient from the study without any form of justification.

If a participant is withdrawn from treatment due to an adverse event, the participant will be followed and treated by the principal investigator until the abnormal parameter or symptom has resolved or stabilized.

All participants who discontinue study treatment should come with their parents/guardians for an early discontinuation visit as soon as possible and then should be encouraged to complete all remaining scheduled visits and procedures. Reasonable attempts will be made by the investigators to provide a reason for participant withdrawals. The number and overarching reason for discontinuation or removal of data will be reported in publication of results.

#### Replacement of participants

Participants who withdraw from the study treatment will not be replaced.

#### Handling of protocol violation

A protocol violation occurs when the participant or investigators fails to adhere to significant protocol requirements affecting the inclusion, exclusion, participant safety, and primary objectives. Protocol violations for this study include but are not limited to the following: Failure to meet inclusion/exclusion criteria; use of a prohibited concomitant medication; or failure to comply with Good Clinical Practice guidelines will also result in a protocol violation. Number and type of protocol violation will be reported in publication of results.

## Discussion

In this randomized blinded controlled trial, we aim to study HR to improve communication between patient and caregiver. This clinical study of HR-informed intervention represents an important contribution to the research and practice of developmental disabilities and communication difficulties. Studies on patients with limited communicative abilities are lacking (Table 4). Existing studies investigating severely limited communicative abilities are small and mainly descriptive. Larger studies on technologies for patients with communication disability exclude the most severely debilitated patients. Furthermore, considerable heterogeneity of participant level of communication disability makes for less clinically relevant findings. In the present study we strive to correct this.

**Table 4:** Methodological advances of current trial relative to past sensor-technology intervention trials among patients with communication difficulties

Limitations in literature^a^	Current study features
Few controlled trials	Randomized controlled design
Small sample sized/insufficient power/case studies	A sample of 38 patients allowing us to detect an 0.80 effect size
Absence of studies using comparator interventions	Controlling for effect of study participation by including a group with delayed intervention
Exclusion of most vulnerable patients	Inclusion of profoundly disabled patients
Lack of standardized measures and biomarkers	HR, HRV and biochemical biomarkers in blood
No adherence data	Routine signing of protocol by secondary participants to measure adherence
Considerable heterogeneity of participants regarding type and level of communication need	Patients with ID ranging from severe to profound and ranging from ambulant to wheelchair user, loss of eyesight and hearing

^a^Based on published reviews [29, 96], and review of studies on PubMed and Google Scholar using the phrases “sensor-technology intervention intellectual disability” and “technology intervention communication difficulties”

Some limitations of the present study design should be noted. *First*, HR responses to events are not a specific measure of pain per se [30], and thus must be interpreted in the situational context to be meaningful. However, four potentially painful situations are predetermined, and based on our pilot [47] there is reason to believe a pattern of HR-increase across situations will become evident over a two week period. Furthermore, to bolster the certainty of pain, we complement the HR measures with other extensively studied biomarkers of pain, such as HRV and pain-related cytokines. *Second*, the caregiver is an intermediary between the researchers and the patient. This means we have no direct objective way to classify situations as all reports of context are given through the caregivers. However, caretakers working with this patient group in Norway are generally well educated and have extensive experience [90], indicating high quality of the observations that are made. We recognize that this indirect method of obtaining contextual information may introduce subjectivity and potential bias. However, we believe that testing the technology through the caregivers is vital to achieving our overarching goal to improve communication and reduce pain in the patient’s everyday life. Setting the study in the patient’s everyday life provides high external validity and ensures that the results are applicable to real-world scenarios. Therefore, while we acknowledge the limitations of our study, we believe that our approach is appropriate and necessary to achieve our research objectives. *Third*, as the current study targets stressors relevant for each participant, the specific stressors are idiosyncratic. The current design allows for rich individual case data, yet the predefined situations and interventions will provide the benefit of group analysis. This may make the method and design unorthodox and initially more difficult to understand. However, adopting this approach allows for a deeper understanding of the complexity of stressors and interventions as the design permits us to gather exploratory, descriptive, and case-specific data. The current approach provides a unique opportunity to combine the rich individual case data with group trends, leading to a more comprehensive understanding of stressors and interventions.

The ethical implications of this study are complex, particularly regarding the patients’ inability to provide informed consent. The mentioned issue of the caregiver as an intermediary is relevant to the ethical considerations taken when designing the study. An apparent way to bypass the intermediary of the caregiver would be to use video recording in addition to HR. However, HR is generally regarded as less intrusive than video recording and considered more ethically just [97]. If video recordings of caregivers were to be conducted, it would be necessary to obtain informed consent from every caregiver involved, as well as any non-participating patients present in the facility. This would introduce additional ethical considerations related to the proper treatment of human subjects in research. We believe HR is informative yet acceptable, a non-invasive technology, possibly of great beneficence to the patients. Another ethical concern here is the aspect of delayed intervention. Obvious painful situations will be detected during the registration phase, and it is not ethical to postpone making changes in these routines for two or four weeks. This ethical aspect makes for more challenging methodology. However, as these instantaneous adaptations are registered, as well as having a delayed intervention group, results will still be valid for evaluation of HR as a communicative aid.

In conclusion, this trial is a randomized blinded controlled clinical trial that test the applicability of HR to reduce incidence of pain in the participant’s everyday life. We will evaluate how HR can be used to identify potentially painful care procedures that should be re-evaluated in terms of the approach taken; test the effect of HR-informed changes in potentially painful care procedures on biomarkers of pain; and assess how six weeks of communication through HR affects the quality of communication between patient and caregiver. Regardless of outcome, the current study will advance the field of wearable physiological sensor-use in patient care.

### Trial status

Inclusion to the study is starting 27th of February 2023. We aim to enrol 38 participants by 2025. The end of data collection will be the end of 2025. Protocol version 1, February 2023.

## Data Availability

Not applicable.

## Abbreviations

ID: Intellectual disability
ASD: Autism spectrum disorder
HR: Heart rate
HRV: Heart rate variability
IQ: Intelligence quotient
NCPC: Non-communicating Children's Pain Checklist
NCAPC: Non-Communicating Adult Pain Checklist
CRP: C-reactive protein
TSD: Services for sensitive data
SD: Standard deviations
ECG: Portable electrocardiogram
EEG: Electroencephalogram
